# Study of the outcome of suicide attempts: characteristics of hospitalization in a psychiatric ward group, critical care center group, and non-hospitalized group

**DOI:** 10.1186/1471-244X-10-4

**Published:** 2010-01-12

**Authors:** Kaoru Kudo, Kotaro Otsuka, Jin Endo, Tomoyuki Yoshida, Hisayasu Isono, Takehito Yambe, Hikaru Nakamura, Sachiyo Kawamura, Atsuhiko Koeda, Junko Yagi, Nobuo Kemuyama, Hisako Harada, Fuminori Chida, Shigeatsu Endo, Akio Sakai

**Affiliations:** 1 Department of Neuropsychiatry, school of Medicine, Iwate Medical University, 19-1, Uchimaru, Morioka, 020-8505, Japan; 2 Department of Critical Care Medicine, school of Medicine, Iwate Medical University, 19-1, Uchimaru, Morioka, 020-8505, Japan

## Abstract

**Background:**

The allocation of outcome of suicide attempters is extremely important in emergency situations. Following categorization of suicidal attempters who visited the emergency room by outcome, we aimed to identify the characteristics and potential needs of each group.

**Methods:**

The outcomes of 1348 individuals who attempted suicide and visited the critical care center or the psychiatry emergency department of the hospital were categorized into 3 groups, "hospitalization in the critical care center (HICCC)", "hospitalization in the psychiatry ward (HIPW)", or "non-hospitalization (NH)", and the physical, mental, and social characteristics of these groups were compared. In addition, multiple logistic analysis was used to extract factors related to outcome.

**Results:**

The male-to-female ratio was 1:2. The hospitalized groups, particularly the HICCC group, were found to have biopsychosocially serious findings with regard to disturbance of consciousness (JCS), general health performance (GAS), psychiatric symptoms (BPRS), and life events (LCU), while most subjects in the NH group were women who tended to repeat suicide-related behaviors induced by relatively light stress. The HIPW group had the highest number of cases, and their symptoms were psychologically serious but physically mild. On multiple logistic analysis, outcome was found to be closely correlated with physical severity, risk factor of suicide, assessment of emergent medical intervention, and overall care.

**Conclusion:**

There are different potential needs for each group. The HICCC group needs psychiatrists on a full-time basis and also social workers and clinical psychotherapists to immediately initiate comprehensive care by a medical team composed of multiple professionals. The HIPW group needs psychological education to prevent repetition of suicide attempts, and high-quality physical treatment and management skill of the staff in the psychiatric ward. The NH group subjects need a support system to convince them of the risks of attempting suicide and to take a problem-solving approach to specific issues.

## Background

General hospitals with an advanced critical care center along with a psychiatry emergency department and a psychiatry ward are annually visited by large numbers of those attempting suicide. They play central roles in treating those who have attempted suicide. Suicide attempters are, after treatment in the emergency room, either hospitalized or sent home. In the case of hospitalization, the attempter will be hospitalized either in a critical care center or in a physical or mental ward.

Concerning outcome, in many instances suicide attempters are instructed to visit the psychiatry department within a few days and are sent home if their condition is mild physically and mentally; they will otherwise be hospitalized in the critical care center if they need to be managed physically in the hospital, or in a psychiatry ward if they need to be managed mentally rather than physically. Apart from such a fundamental policy, suicide attempters often present with various conditions both physically and mentally, which, in emergency situations, should be properly dealt with in an appropriate facility.

Chiles, J. A. and Strosahl, K. D. indicate that it is imperative to address the problem of "voluntary or involuntary psychiatric hospitalization" in treating suicidal risk [1]. In treating patients with suicidal behavior, they believe it is important "to closely monitor reinforcement patterns on the unit so that suicidality is not being exacerbated." Baca-García, E, et.al. (2004) suggest placing top priority on "the guidelines for assessing suicide attempts need to encourage thorough and detailed assessment of the attempt and the future plan" in determining whether suicide attempters who visited the critical care center should be hospitalized or not [2].

In the present circumstances, however, these types of responses are not performed, or current situation has not been reviewed due to a lack of extensive data.

In this study, we categorized suicide attempters treated in the emergency room into three groups - those who were hospitalized in the critical care center, those who were hospitalized in a psychiatry ward (presently closed), and those who were sent home - and examined each group's characteristics (i.e., background factors such as sex and age, psychiatric diagnosis and medical history, and methods of suicide attempt) and the severity and differences among groups. Logistic regression analysis was then performed to examine predictors of each outcome. The purpose of this study was to examine, from the perspective of outcome, how suicide attempters are allocated as well as to identify the potential needs of each outcome group.

## Methods

A total of 10,020 cases at the Critical Care and Emergency Center ("the Center") and the psychiatry emergency department of Iwate Medical University Hospital during the period between April 1, 2002 and March 31, 2008 were considered psychiatric emergency cases. Of them, 1,434 involved suicide attempts, and after excluding 86 cases of patients who had died or had been referred to other hospitals, we examined the remaining 1,348 cases (Additional file 1, Table 1).

Following categorization of suicidal attempters by outcome, into the HICCC group hospitalized in the advanced critical care center, the HIPW group hospitalized in the psychiatry ward (presently closed) of Iwate Medical University Hospital, and the NH group sent home, we examined a total of twenty items for each group, including sex, age, years of education, living status, work status, first/return presentation to psychiatry, consultation prior to suicide attempt, number of episodes of depression in lifetime, history of suicide-related behavior (lifetime and during the past year), and items for diagnostic classification of mental and behavioral disorders according to the International Statistical Classification of Diseases and Related Health Problems: 10^th ^Edition ("ICD-10") [3]. In addition, for evaluable patients, we used the Brief Psychiatric Rating Scale (BPRS) of the Oxford University Version (translated by Kitamura, et al.) [4] to evaluate psychiatric symptoms as well as the Global Assessment Scale (GAS)(translated by Kitamura, et al.)[5]to examine overall psychiatric symptoms and daily life capacities. In addition, we assessed life events prior to suicide attempts, such as spouse's death and debts, using Life Change Units (LCU) [6] of the Holms Social Readjustment Rating Scale.

The physical severity of each suicide attempts was assessed using Asukai's Criteria [7]. These criteria adopted for the classification of the absolutely dangerous group (AD group) were as follows: jumping from a height (>10 m), jumping in front of a moving train, cutting or stabbing internal organs, hanging, drug overdosing or other poisoning, requiring medical attention (e.g. mechanical respirator, hemodialysis), severe burning, gassing, and drowning. All subjects were divided into two groups: the AD group and the relatively dangerous group (RD group).

It has been pointed out that, in emergency situations, it often becomes difficult to understand or record clinical information[8-10]. Since 2000, we have used case cards to record the patient's demographic information, psychiatric assessment, prognosis, and other treatment information, obtained from the patient, his/her family, and the rescue crew, for all patients treated by psychiatric emergency doctors (1,400 cases per year). The 1348 cases assessed in this study were recorded in the same fashion. Assessment and diagnosis for each item were conducted by eight psychiatric emergency physicians or doctors on duty at the University Hospital, under the supervision of a senior psychiatrist (the designated psychiatrist). Management and processing of the data were performed so as to ensure the protection of personal information, and personally identifiable items were excluded from the data.

SPSS 15.0 J for Windows was used for statistical processing. One-way analysis of variance was used for comparing mean values of three groups, the Bonferroni method for mean values of two groups, and the χ^2 ^test for ratios (Additional file 1, Table 1 and Additional file 2, Table 2). For items exhibiting significant differences, multiple logistic analysis was performed to extract outcome-related factors, considering test items as explanatory variables and "hospitalization in the psychiatry ward" (yes = 1, no = 0), "hospitalization in the Center" (yes = 1, no = 0), and "non-hospitalization" (yes = 1, no = 0) as dependent variables (Additional file 3, Table 3). In every test, the significance level was 5%. Probabilities of significance are shown in tables.

### Approval of the study protocol

The study protocol was reviewed and approved by the Research Ethics Committee of Iwate Medical University, School of Medicine.

## Results

### 1. Background Factors

#### Additional file 1

The HIPW group (N = 486, male; 160) had the highest number of cases, followed by the HICCC (N = 475, male; 209) group and the NH group (N = 387, male; 48) in this order. There were significant differences in the percentage of males among the three groups (p < 0.001), and the percentage of males was highest in the HICCC group. There were significant differences in average age among the three groups (p < 0.001), and the percentage was highest in the HICCC group, followed by the HIPW group and the NH group as determined by the Bonferroni test conducted later. There were significant differences in the percentage of first and second visits among the three groups (p < 0.001), and the HICCC group exhibited the highest percentage at 64.2%, while both the NH group and HIPW group had about 50%. There were significant differences in the modality of hospital presentation among the three groups (p < 0.001), and most of the HICCC group and many of the HIPW group patients were tertiary outpatients. Finally, there were also significant differences in psychiatric consultation history among the three groups (p < 0.001); the percentage of subjects with a history of such was higher in the NH and HIPW groups than in the HICCC group.

### 2. Clinical Rating, Diagnosis, Method of Suicide Attempt, and Regimen

#### Additional file 2

There were differences among the three groups in ICD-10 diagnoses. In the NH group, F4 (Neurotic, stress-related and somatoform disorders) was highest (48.1%), followed by F3 (Mood disorder; 23.8%), while in the HICCC group F3 was the highest (37.1%) followed by F4 (25.9%). In the HIPW group, F4 (32.5%) and F3 (30.9%) were nearly the same, and accounted for more than half of all diagnosis.

In severity of disturbance of consciousness (JCS) (p <  0.001) and general health performance (GAS average) (p < 0.001), significant differences were recognized among the three groups, with JCS and GAS, highest in the HICCC group, followed by the HIPW group and then the NH group. There were significant differences among the three groups in psychiatric symptoms (total BPRS) (p = 0.001) and life events (average LCU) (p < 0.001). In addition, the score was highest in the HICCC group, followed by the HIPW group and NH group (Bonferroni-test). A significant difference was recognized between the NH group and the HICCC/HIPW groups in BPRS and LCU, though not between the HICCC group and the HIPW group.

There were also significant differences among the three groups in method of psychotherapy, psychotropic agent administration, physical treatment, internal use of psychotropic drugs, and psychotropic drug injection (p < 0.001). Among methods of suicide attempt, drug overdose was most common in all three groups. In the NH group, cutting and overdosing accounted for more than 80% of cases. In the HIPW group, the proportion of cases of cutting was slightly lower than in the NH group, while many serious methods, such as gassing and drowning, were also used, though not in the NH group. Compared with other two groups, the HICCC group used a greater variety of methods, including poisoning, gassing, jumping, and burning in particular, which could have serious physical sequelae.

Treatments provided in the emergency room also differed among the three groups. In the NH group, more psychotherapy and psychotropic agents were administered but less physical treatment was administered compared with other two groups. In the HICCC group, in contrast, more physical treatment was administered and less psychotherapy and fewer psychotropic agents were administered.

### 3. Logistic Regression Analysis

#### Additional file 3

To extract factors related to outcome after treatment in the emergency room, we performed logistic regression analysis among the three groups. The analysis was carried out with age, years of education, total score of BPRS, average GAS score, average LCU score, JCS score, sex, first/return visit, previous psychiatric history, history of suicide-related behavior in lifetime, history of suicide-related behavior within the past year, treatment provided in the emergency room, ICD diagnosis, and method of suicide attempt as explanatory variables. As a result, the following nine items were extracted as outcome-related factors: age, BPRS, GAS, JCS, sex, first/return visit, history of suicide-related behavior, method of suicide attempt, and treatment provided in the emergency room.

The odds ratio for the NH group increased 0.987 (p =  0.033) with one year increase in age, as well as 0.979 (p = 0.015) in BPRS, 1.010 (p = 0.015) in GAS, and 0.986 (p < 0.001) in JCS. The odds ratio for men was 0.311 (p < 0.001) compared to women, that for the delivery of physical treatment compared to absence of it 0.460 (p < 0.001), that for the delivery of psychotherapy compared to the absence of it 1.680 (p = 0.002), and that for psychotropic agent administration compared to the absence of it  12.217 (p = 0.035).

In the HIPW group, the odds ratio was 1.462 (p = 0.011) for men compared to women, while that for JCS was 0.997 (p < 0.001). The odds ratio for the delivery of suicide-related behavior over a lifetime compared to the absence of it was 0.643 (p = 0.020), while by method of attempted suicide it was 0.092 (p < 0.001) for drug overdose, 0.203 (p = 0.018) for gassing, 0.251 (p = 0.045) for jumping, and 0.030 (p = 0.004) for burning.

In the HICCC group, the odds ratio was 1.016 (p = 0.003) for age, 1.022 (p = 0.010) for BPRS, and 1.008 (p < 0.001) for JCS. The odds ratio was 1.544 (p = 0.011) for men compared to women, that for first visit compared to return visit 1.504 (p = 0.014), that for the delivery of physical treatment compared to the absence of it 2.957 (p < 0.001), and that for the delivery of psychotherapy compared to the absence of it 0.333 (p < 0.001), while by method of attempted suicide it was 21.351 (p = 0.007) for overdose, 11.733 (p = 0.034) for gassing, 21.671 (p = 0.007) for jumping, and 78.022 (p = 0.005) for burning.

## Discussion

### 1. Sex, Age, and Modality of Hospital Presentation

Previous reports pointed out that, globally, suicidal attempts are more common in women, while suicide-related behaviors by men tend to be more serious, resulting in completed suicides in many cases [11,12]. Psychologically speaking, in some cases, suicide-related behaviors do not always mean that attempters would like to die, but they function as an unconscious signal for help. Such help-seeking behaviors are particularly notable in women, and used to be termed "parasuicides," [13,14] however, they are termed "deliberate self harm" in the extant literature. In this study, as well, there were more women than men among those who visited the emergency room due to a suicide attempt, and more than 80% of the NH group patients were women. It is presumed that, in the case of deliberate self harm, which is more common among women, many suicide attempters stop short of hospitalization, since the intention of suicide is unclear and they only receive minor injuries.

According to studies on the outcomes of suicide attempts, including completed suicides, the ratio of men is highest in the "completed suicide" group, then in the hospitalized group, and lowest in the outpatient group [15]. It is more likely that, compared to women, men do not consult with the people around them prior to suicide attempt and often refuse to see a psychiatrist, even if the people around them notice changes and encourage them to do so [16]. In this study, the same tendency was observed as in previous studies, since the ratio of men was highest in the HICCC group and next highest in the HIPW group. It is presumed that men tend to have too much stress themselves without consulting the people around them, and develop psychological tunnel vision [17], causing more serious physical conditions because they seek more certain means of death.

High suicide rates among the elderly are commonly observed in advanced countries, and it is pointed out that the cause of this is partly related to depression [18]. Also, regarding those who attempted suicide without success by highly life-threatening means, the presence of depressive disorder was often recognized among patients over 50 years of age [19]. It was also reported in the outcome survey of suicide attempters noted above that the age of suicide attempters is higher in the hospitalized group than in the outpatient group, and is again higher than in the completed suicide group than in the hospitalized group [15]. In this study, average age was the highest in the HICCC group, next highest in the HIPW group, and lowest in the NH group. This may reflect the fact that the elderly tend to have more physical co-morbidity and stress events, such as the experience of loss.

By modality of hospital presentation, many tertiary outpatients transported by ambulance were found in the hospitalized group. They were taken by ambulance due to serious physical conditions. On the other hand, it is also likely that the suicide attempters themselves and the people around them were concerned enough to call for ambulance and that they strongly desired that the patient be hospitalized. Therefore, even in cases in which after examination and treatment in the emergency room it is judged that hospitalization is not medically warranted, it will be required to provide appropriate and sufficient psychotherapy and detailed explanation of no need for hospitalization.

### 2. ICD Diagnosis, Previous Psychiatric History, and Suicide-Related Behaviors

Psychiatric disorders are regarded as risk factors for suicide [20-24], and the importance of F3 and F4 in this respect has been pointed out in particular. In a comparison between F3 and F4, among suicide-related behaviors, it was reported that many severe methods of suicide-attempt were found in F3 [25]. In this study, as well, F3 was most commonly observed in the HICCC group, suggesting the effects of serious physical conditions resulting from severe methods of attempted suicide.

In addition, the ratio of F2 (Schizophrenia, schizotypal and delusionaldisorders)patients was higher in the hospitalized group than in the NH group. The causes of suicide in schizophrenics presently include extraordinary experiences, such as hallucinations due to reactivation, and depression resulting from problems with social life [19]. Also, compared with other psychiatric patients, even if those with schizophrenia tell others their intention to commit suicide, it is often overlooked as part of their psychiatric condition and is not recognized as a suicidal tendency [26]. It is anticipated that difficulty in predicting suicide attempts may exacerbate hallucinations and depression, causing physically and mentally severe conditions that may even require inpatient hospital care.

It is pointed out that many patients with completed suicide had not visited any psychiatric institution prior to their suicidal behavior [27,28]. It is also reported that, in the "absolutely danger (AD)" group, which Asukai, et al. say exhibits more severe physical conditions associated with suicidal attempts, there are many patients who first visited a psychiatric institution or cases which patients tried to commit suicide for the first time [29]. In this study, it was found that about 50% of the NH group and the HIPW group, in addition to about 60% of the HICCC group, were first-visit patients, and that suicide-related behaviors were most common in the NH group, next most in the HIPW group, and least common in the HICCC group, suggesting that first suicide attempts tend to be associated with more physically serious conditions.

These findings indicate the likeliness of making a suicide attempt as a result of exacerbation of psychiatric disorder if the patient him/herself or the people around him/her do not notice the potential for such and the patient refuses to see a psychiatrist; or worse, the risk of causing more serious physical problems if a suicide attempt is made without treatment, with more severe methods.

It is therefore important to increase opportunities to raise the awareness of community residents of the importance of preventing suicides as well as detecting mental disorders, such as depression, even in medical institutions other than psychiatry departments. On the other hand, among deliberate self harm cases, who have exhibited suicide-related behaviors several times and who do not have physically serious conditions, and among those whose suicidal feelings were temporarily weakened after an attempt due to its cathartic effect [30], it is very likely that attempts will be repeated, finally with a higher rate of fatality [31-33]. Even if the patient is judged safe enough to go home after outpatient treatment, it is necessary to determine the process by which he/she came to try to kill him/herself, and to provide careful treatment, such as introduction of proper psychotherapy or encouragement to visit a psychiatrist in the future.

### 3. Methods of Suicide Attempt, Outpatient Treatment, and Physical/Mental Severity

Methods of suicide attempt vary by the country; however, hanging is most common throughout the world. It is reported that men use guns and women prefer drug overdose [12]. In this study, drug overdose was most common in all three groups. We believe that this is because these groups included large numbers of female subjects. In a survey previously conducted, we found that, in the mild "Relatively Danger" group (Asukai) [7], often found in the NH group, the majority of the methods used involved either drug overdose with low fatality or impulsive wrist cutting just on the skin surface, without any clear intention of ending life [29]. In the present study, it was found that approximately 80% of methods used in the NH group involved knives and drug overdosing, and it is believed that many similar cases were included in the NH group.

In the HICCC group and the HIPW group, a variety of methods, which were often severe, were used. In the HICCC group, many dangerous methods with high fatality were employed, and the ratio of administration of physical treatment was higher than in the other two groups. On the other hand, the ratio of provision of psychiatric treatment was about 10%. We believe early psychiatric intervention is necessary in such cases, as it is believed that the choice of method is related to the strength of suicidal feeling.

Concerning JCS scores, it was confirmed that both state of consciousness and the severity of physical condition strongly affect outcome. In particular, patients with poor state of consciousness or patients with physically severe conditions that require physical control are certainly indicated for hospitalization in the Center. Significant differences were recognized among the three groups in terms of GAS as well as between the NH group and the other two groups in terms of BPRS, though no significant difference was recognized between the HIPW group and the HICCC group in BPRS. It is believed that the presence or absence of physical conditions determines where the patient should be hospitalized, since physical conditions are included in GAS but not in BPRS items.

A significant difference was recognized between the NH group and the HICCC group in LCU. It is suggested that accumulation of life events causes the risk of making more physically-serious suicidal.

### 4. Multiple Logistic Regression Analysis

Risk factors for the NH group, NIPW group, and HICCC group were identified by multiple logistic regression analysis. Spearman's correlation coefficients among the three outcome categories as well as items with a large confidence interval, i. e., taking psychotropic drug, poisoning, gassing, jumping and burning, were between -0.200 and 0.041. It thus appeared that there were no marked effects of multicollinearity on those findings with a large confidence interval.

In a previous study, Gaca-García, E. et al (2004) listed the following as causes for increased odds ratios of hospitalization for suicide attempters who visited the critical care center: intention to repeat the attempt, plan to use a lethal method, low psychosocial functioning before the suicide attempt, previous hospitalization, a suicide attempt within the past year, and planning that nobody would try to save their life after they had attempted suicide[2]. They also listed causes for decreased odds ratios as follows: a realistic perspective on the future after the attempt, relief that the attempt was not effective, availability of a method to kill oneself (that was not used), belief that the attempt would influence others, and family support.

In our results, the extracted factors that increased risk of hospitalization in a critical care center were higher age, higher BPRS/JCS scores, male sex, first presentation, delivery of physical treatment, absence of psychotherapy, and suicide methods such as poisoning, gassing, and burning.

On the other hand, the factors which increased the risk of hospitalization in the psychiatric ward were lower JCS scores, male sex, and absence of suicide-related behaviors over the lifetime, while those which decreased the risk were suicidal methods such as poisoning, gassing, jumping, and burning.

Also, the factors related to non-hospitalization were lower age, lower BPRS/JCS scores, higher GAS scores, female sex, delivery of psychotherapy, use of psychotropic drugs, and absence of physical treatment.

Summarizing the results, it appears that the severity of disturbance of consciousness or suicide methods, that is, the severity in physical conditions, affects the choice of care setting. It also appears that the type of emergency care provided at the time of visit, that is, whether or not physical treatment was administered or psychotherapy was performed, affects choice of treatment. Needless to say, it should be noted that, since the HICCC group was in general severely injured physically with impairment of consciousness, psychiatric treatment was hardly offered to them. Interestingly, it was found that risk factors for suicide, i.e. sex, history of suicide-related behaviors, and severity of psychiatric condition, affected the choice of care setting. It appears that assessment of the risk of suicide directly affects the choice of treatments for suicide attempters.

In conclusion, it was found that, in the care for those attempting suicide, the severity of physical conditions, risk factors, assessment of emergent medical intervention, and the type of care provided were strongly related to hospitalization in a critical care center, hospitalization in the psychiatric ward, or non-hospitalization.

### 5. The Potential Needs of Patients in Each Outcome Group

Previous studies reported that, while patients with schizophrenic hallucinations or depression caused by schizophrenia should be hospitalized and treated as inpatients, those with increased impulsiveness and impaired judgment caused by alcohol etc. can be treated as regular outpatients with supportive psychotherapy and crisis intervention [34]. Also, there is a proposal for management of suicide attempters according to which those who have psychiatric problems as a cause of suicide attempt are indicated for hospitalization if there is a risk of repetition of the attempt or harming others, while those who have serious physical conditions should be referred to the general emergency room [35]. In addition, strength of suicidal feeling is listed as one of the important items of evaluation in judging the outcome of suicide attempts at the scene of the emergency [36]. Some foreign studies report that men of 45-years of age or over who have a newly developed psychiatric problem and strong suicidal feeling with fatal method should be hospitalized if they are not in the supportive environment, while those who have chronic suicidal feeling and are under psychiatric treatment in a supportive environment with no fatal method can be effectively treated as outpatients [37].

The previous studies noted above considered allocation of outcome according to psychiatric diagnosis, strength of suicidal feeling, support system, and severity of method. In this study, it was found that the hospitalized groups, compared to the NH group, had more serious disturbance of consciousness (JCS), poorer mental, physical, and social health performance (GAS), more severe psychiatric conditions (BPRS), and relatively significant life events (LCU). It was also found that, among the hospitalized patients, those who were hospitalized in a critical care center were in worse condition than those hospitalized in the psychiatric ward.

As a result, it was found that the outcome of suicide attempts is affected more by the severity of physical, mental, and social conditions than diagnostic classification, and that the HICCC group is composed of patients who has more serious problems physically, mentally, and socially.

Considering the serious problems this group faces, it is clear that biopsychosocial care should be started immediately by incorporating psychiatric treatment in the physical emergency care system. Specifically, psychiatrists should be stationed in the critical care center on a full-time basis for early psychiatric intervention, and, based on that system, social workers and clinical psychotherapists should be introduced to be partnered with social resources. In other words, it can be pointed out that serious suicide attempters should receive comprehensive care by a medical team composed by multiple professionals at the critical care center.

Concerning those hospitalized in the psychiatric ward, it should be noted that they have less serious physical conditions but cannot be discharged psychiatrically. It is necessary to improve inpatient psychiatric treatment and practice psychological education to prevent repeat attempts, since psychiatric disorders and suicide attempts are strongly related. It is also expected to improve the level of physical treatment and management skill of the staff in the psychiatric ward.

Finally, most of the NH group members were women, who tend to repeat suicide-related behaviors triggered by relatively small stressors. This group did not need to be hospitalized, with administration of psychotherapy and physical treatment at the time of emergency visit. It cannot be denied, however, that the members of this group might repeat attempts and complete suicide in the future. Some reports indicate that attempted suicide is a risk factor for completed suicide [31-33] and the major risk factor for repeat attempts is co-morbidity with psychiatric conditions [38]. In order to prevent repeat attempts, it is necessary to rapidly establish a support system to encourage patients to see a psychiatrist after the emergency visit, to confirm the risk of committing suicide, to take a psychotherapeutic approach to improve coping with stress, and to take a problem-solving approach to specific issues. To realize such a support care system, it is essential for emergency medical care, community medical care, and community psychic health care services to work hand-in-hand.

## Conclusions

We aimed to identify the characteristics and potential needs of 3 groups, i.e., hospitalization in a psychiatric ward group (HIPW group), critical care center group (HICCC group), and non-hospitalized group (NH group). The HICCC group needs psychiatrists on a full-time basis and also social workers and clinical psychotherapists to immediately initiate comprehensive care by a medical team composed of multiple professionals. The HIPW group needs psychological education to prevent repetition of suicide attempts, and high-quality physical treatment and management skill of the staff in the psychiatric ward. The NH group subjects need a support system to convince them of the risks of attempting suicide and to take a problem-solving approach to specific issues.

## Competing interests

The authors declare that they have no competing interests.

## Authors' contributions

KK and KO analyzed the data and wrote the paper. AS supervised and wrote the paper. JE, TY, HI, TY, FC assessed the patients. HN participated in the design of the study and performed the statistical analysis. SK, AK, JY, NK, HH participated in the study as a whole and commented on the manuscript. SE conceived of the study, and participated in its design and coordination. All authors approved the final manuscript.

## Pre-publication history

The pre-publication history for this paper can be accessed here:

http://www.biomedcentral.com/1471-244X/10/4/prepub

## Supplementary Material

Additional file 1 **Table 1. Background Factors**. Background factors of the subjects, i.e., sex, mean age, average years of education, living status, status of work, hospital presentation modality, first or return presentation, previous psychiatric history, history of suicide-related behavior within the past year, history of suicide-related behavior in lifetime, number of episodes of depression, presence or absence of person to consult. Click here for file

Additional file 2 **Table 2. Clinical rating, Diagnosis, Method of Suicide Attempt, and Regimen**. Data of the subjects, i.e., scores of GAS, BPRS, LCU, JCS, ICD-10 diagnosis, method of suicide attempt, presence or absence of psychotherapy, psychotropic agent administration, physical treatment, internal use of psychotropic drug, psychotropic drug injection. Click here for file

Additional file 3 **Table 3. Multiple Logistic Regression**. background factors and clinical rating, diagnosis, method of suicide attempt, and regimen. Click here for file
